# New Appliances and Things Medical

**Published:** 1899-06-03

**Authors:** 


### NEW APPLIANCES AND THINGS MEDICAL.

[We shall be glad to receive, at our Office, 28 & 29, Southampton Street, Strand, London, W.C., from the manufacturers, specimens of all new preparations and appliances which may be brought out from time to time.]

### ASEPTIC SPONGES.

(SQUIRE AND SONS, 413, OXFORD STREET, W.)

THIS new form of sponge strikes us as particularly ingenious and useful. It consists of cotton fibre subjected to great pressure about the size of a five-shilling piece, and one-twelfth of an inch in thickness. When placed in water it swells to fifteen times its size, and will absorb about twelve times its weight of fluid. For minor surgery they should prove of value. For instance, in tamponing the anterior or posterior nares they need be less frequently changed than less absorbent forms of sponge or plug. For ordinary toilet use they would certainly prove a cleanly and convenient substitute for the ordinary sponge. The sponges are rendered aseptic before being packed in aseptic boxes.

### SQUIRE'S CHEMICAL FOOD LOZENGES.

(SQUIRE AND SONS, 413, OXFORD STREET, W.)

THESE lozenges, which are by no means disagreeable and would be readily taken by children, are a convenient form in which to give Squire's well-known chemical food or Syrup-ferri-phosphat. Co. Each lozenge contains an equivalent of about half a teaspoonful of the syrup. The idea is a most ingenious one, and as far as we can ascertain the therapeutic nature of the preparation undergoes no deterioration by the process.

### MIGROL. GUAJACETIN.

(THE ANGLO-CONTINENTAL CHEMICAL WORKS, LIMITED, 1 AND 2, RANGOON STREET, LONDON, E.C.)

GUAJACETIN is a synthetic preparation suggested for use as a substitute for guajacol, creosote, and their compounds in the treatment of phthisis. It is chemically described as sodium pyrocatechin mono-acetate. It is supplied in the form of eight-grain tablets, which are readily soluble in warm water, and have a not unpleasantly bitter taste. The great advantage over guajacol and the like preparations is the inodorous character and non-irritating action of guajacetin, whilst it is stated to be absolutely non-poisonous and to possess all the good features of guajacol. Illustrations from Continental practice endorse the view as to guajacetin being an efficacious and perfectly harmless remedy in tuberculous diseases. The dose is eight grains three times daily. Migrol is a combination of guajacetin with a caffeine compound of analogous constitution. It is intended for use as an anodyne in cases of nervous headache, and is stated to be quite innocuous. Like all synthetic preparations for medicinal use, such remedies should only be administered under the advice of medical authority.

### ANTISEPTIC VAGINAL CAPSULES.

(SQUIRE AND SONS, 413, OXFORD STREET, W.)

THE method of applying wool tampons for gynæcological use encased in gelatine capsules is one that has already proved its usefulness. The medicament, whether it be glycerine, iodoform, ichthyol, or other form of antiseptic, can by this method be placed *in situ*, and kept there, without the tampon undergoing the risk of being squeezed nearly dry by the difficulties of insertion. In the particular form of capsules referred to above the covering is composed of specially carbolised gelatine, which readily dissolves at the blood temperature, and adds its own quota of antiseptic properties to the therapeutic effect of the medicated tampon. The tampon has a fine thread attached to facilitate removal after use.

### CHOCOLATE-COATED TABLETS.

(PARKE, DAVIS, AND CO., 21, NORTH AUDLEY STREET, LONDON, W.)

THE chocolate-coated tablets which are now being introduced by Messrs. Parke, Davis, and Co. strike us as a useful and elegant preparation. The coat[illegible]g of chocolate with which they are invested renders them palatable, easily soluble in the stomach, and more permanent. The list of drugs and combinations of drugs which are put up in this form number at present some two hundred, and include those formulæ which have proved most popular in pill or simple tablet form. The tablets, which are specially intended for infants and children, may be crushed and administered in the form of an agreeable powder. Those who are desirous of trying this up-to-date pharmaceutical development should write to the firm for sample preparation.

### NEW PROTECTIVE PACKING.

(HORROCKS AND CO., STANDISH, WIG[illegible]N.)

IN reference to this paper packing, which is [illegible]rimarily intended for the protection of bottles and ot[illegible] breakable articles, the makers inform us that they now [illegible]ke it up in a stronger and heavier quality, faced with coloured paper, in the form of mats for use in sick rooms. It is resilient and warm to the tread. A paper mat which can be destroyed after it has served its purpose is likely to be found very useful in dealing with infectious cases in private houses.

